# Adsorption of Ciprofloxacin onto CMCs/XG Hydrogel: Optimization, Kinetic, and Isotherm Studies

**DOI:** 10.3390/polym18050632

**Published:** 2026-03-04

**Authors:** Sitah Almotiry, Dalal M. S. Almuthaybiri, Nouf F. Al-Harby, Nadia A. Mohamed

**Affiliations:** Department of Chemistry, College of Science, Qassim University, Buraidah 51452, Saudi Arabia

**Keywords:** carboxymethyl chitosan, xanthan gum, hydrogel, ciprofloxacin, adsorption, isotherms, kinetics, thermodynamics

## Abstract

The use of adsorbents based on naturally occurring materials to eliminate antibiotics from industrial effluents has attracted remarkable interest owing to the abundance of raw materials and the sustainability of this method. The ciprofloxacin (CIP) removal capacity of a previously synthesized antimicrobial hydrogel based on carboxymethyl chitosan (CMCs)/xanthan gum (XG) was investigated for the first time in this study. CMCs and XG were blended in an equivalent-weight ratio and crosslinked using trimellitic anhydride isothiocyanate (TAI) to synthesize an eco-friendly, low-cost hydrogel, which was characterized using FTIR, SEM, and XRD analyses. The pseudo-second-order model fitted the experimental data well: the experimental *q_e_* (49.59 mg g^−1^) was close to the theoretical value (51.81 mg g^−1^). The Langmuir isotherm best fitted the adsorption results (*R*^2^ = 0.999), with a maximum adsorption capacity of 147.06 mg g^−1^. The thermodynamic results indicate that adsorption is spontaneous, favorable, and exothermic in nature. The percentages of desorption obtained were 95.72, 94.34, 89.52, 88, and 86.28% after five consecutive cycles. Thus, this hydrogel possesses potential for further testing and application in wastewater remediation.

## 1. Introduction

Among emerging pollutants in aquatic ecosystems worldwide, pharmaceuticals are recognized as hazardous compounds because of their negative impact on the health of aquatic living organisms and humans [1]. Pollution by pharmaceuticals used for medical applications, particularly antibiotics used for treating or preventing diseases, has increased due to their intensive and indiscriminate utilization [2].

Ciprofloxacin (CIP) is an antibiotic drug commonly used in the treatment of many bacterial infections in animals and humans [3]. CIP persists in aquatic resources because it is not completely metabolized by the living organisms in these environments. This leads to a rise in resistance against CIP, treatment failure [4], and CIP accumulation in the food chain [5].

Various techniques have been employed to remove CIP, including electrochemical [6] and chemical [7] oxidation, photocatalytic and photolytic remediation [8], ozonation [9], biological remediation and enzymatic degradation [10], photo-Fenton oxidation [11], and adsorption [12].

In general, adsorption processes have shown excellent efficiency in the removal of various contaminants, such as heavy metals [13,14], synthetic dyes [15,16], and antibiotics [17], from water. In addition, adsorption processes have various advantages, including simplicity, rapid kinetics, ease of implementation, low cost, adsorbent availability, non-harmful by-products, and ease of regeneration of the adsorbents for reuse in further cycles. In this context, several studies on CIP removal from aqueous media have reported the use of different adsorbents, including kaolinite [18], nano-sized magnetite [19], schorl [20], halloysite [21], graphene oxide/calcium alginate [22], montmorillonite [23], synthesized nanoceria [24], and activated carbon [25].

Chitosan (Cs) is a natural cationic polysaccharide usually extracted by partially deacetylating chitin. It possesses numerous hydroxyl and primary amino groups, which can be employed as active binding sites for the adsorption of pollutants. Cs is characterized by abundance, non-toxicity, environment-friendly nature, low cost, high viscosity, film- and gel-forming capacity, biodegradability, biocompatibility, and biological activity. However, it only dissolves in acidic media, which limits its application [26]. Thus, it can be chemically modified into water-soluble derivatives, particularly its ether derivative, carboxymethyl chitosan (CMCs), which shows solubility in a wide range of pH values while displaying the other properties of chitosan [27]. 

Xanthan gum (XG) is a natural anionic polysaccharide produced by the Gram-negative bacterium Xanthomonas campestris. It is extensively utilized in different fields, including the food, textile, biomedical, cosmetic, pharmaceutical, and petrochemical industries, and is biodegradable, biocompatible, thermally stable, and non-toxic. However, a significant shortcoming restricts its potential applications: it is susceptible to microbial contamination when degraded under microbial attack [28,29,30].

Thiourea-based CMCs has shown effective adsorption characteristics with dyes [31,32] and metals [33], ascribed to the synergism between the sulfur and nitrogen atoms of thiourea and the functional groups of CMCs. This allows for strong interactions with CIP, including electrostatic and H-bonding under specific pH conditions [34].

Consequently, it is expected that inducing thiourea-based crosslinkages between CMCs chains by using trimellitic anhydride isothiocyanate (TAI), as well as leveraging the hydroxyl and carboxylic groups in both XG and CMCs, can increase the number of active sites available to bind the antibiotic CIP. Furthermore, the crosslinking of CMCs based on TAI could improve the former’s characteristics, increase its functional groups, lower its solubility, inhibit its degradability, and increase its product life span in various media, thus facilitating its regeneration for further reuse.

Therefore, CMCs and XG (in a 1:1 weight ratio) were blended and then crosslinked using TAI as shown in our previous research work [35]; the obtained CMCs/XG hydrogel was investigated as an adsorbing material to remove CIP from aqueous solutions for the first time in this study. To optimize the adsorption conditions, the influence of the variables that govern adsorption capacity, i.e., the dose of hydrogel, the initial concentration of CIP, the pH of the solution, temperature, and time, was evaluated. The kinetics, isotherms, and thermodynamics of the process of CIP adsorption onto the hydrogel were studied. Finally, the viability of regenerating the hydrogel for reuse was also investigated.

## 2. Chemicals and Methodology

### 2.1. Chemicals

**Figure 1:**
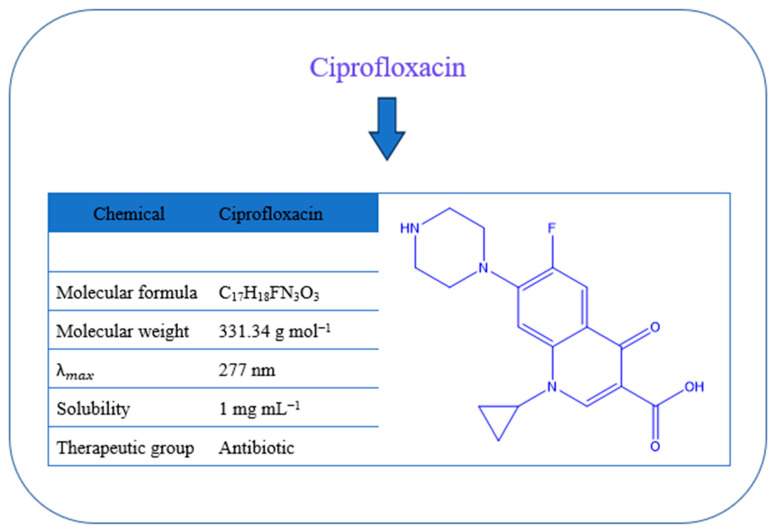
Antibiotic CIP: chemical structure and characteristics.

**Figure 2:**
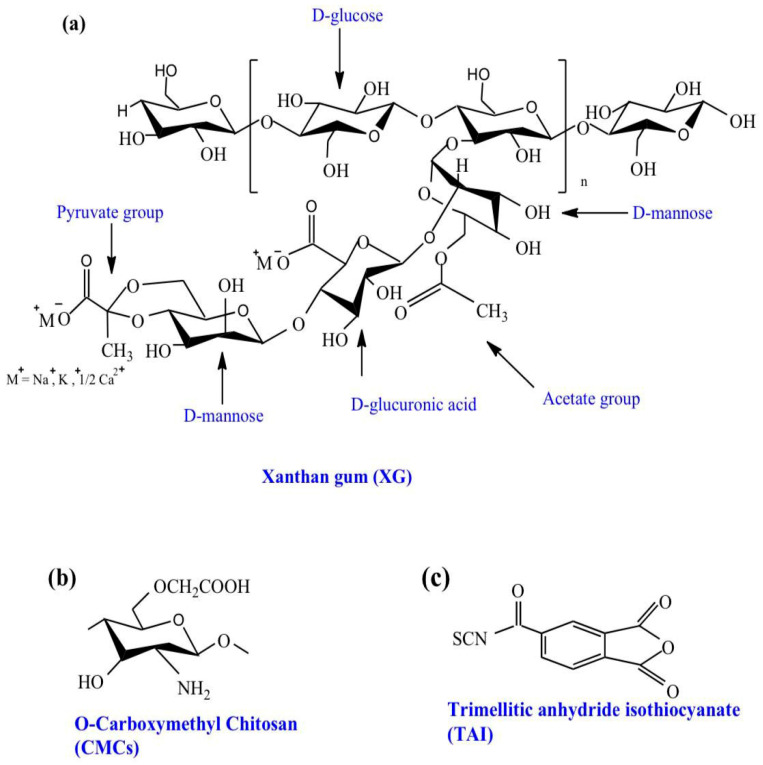
The chemical structures of (**a**) XG; (**b**) CMCs; and (**c**) crosslinker (TAI).

The antibiotic ciprofloxacin (CIP; ≥99% purity; Figure 1) was supplied by SPIMACO (Dammam, KSA). Chitosan (Cs) extracted from *pandalus borealis* crab and shrimp shell was purchased from Across Organics (Fair Lawn, NJ, USA). It had a molecular weight of 100,000–300,000 g/mol and a deacetylation grade of 0.98. Xanthan gum (XG; originating from *Xanthomonas campestris*; Figure 2a), trimellitic anhydride chloride (98%), polyethylene glycol (PEG 400), chloroacetic acid (99%), and ammonium thiocyanate (99%) were obtained from Sigma-Aldrich (Munich, Germany).

### 2.2. Methods

#### 2.2.1. O-Carboxymethyl Chitosan (CMCs) Preparation

CMCs (Figure 2b) was prepared in accordance with a previously described procedure [36]: Cs (5 g) was stirred in isopropanolic NaOH solution containing five droplets of distilled water (7 g, 50 mL) at room temperature for 45 min to make it swell and alkalize. Monochloroacetic acid (7.5 g) was slowly introduced into the mixture and stirred well at 328 ± 5 K for four hours. Afterwards, the alkaline medium was treated with an aqueous solution of acetic acid (20% *v*/*v*) to obtain pH 7. Then, isopropanol was discarded, and 300 mL of aqueous solution of ethanol (85%) was introduced to precipitate the formed CMCs. The precipitated CMCs was obtained by filtering and then washing it repeatedly using the aqueous solution of ethanol (85%) to desalt it and dewater it; finally, it was dried at 343 ± 5 K (7.5 g). The CMCs substitution degree was found to be 80%, in accordance with the previously described method [37].

#### 2.2.2. Synthesis of CMCs/XG Hydrogel

**Figure 3:**
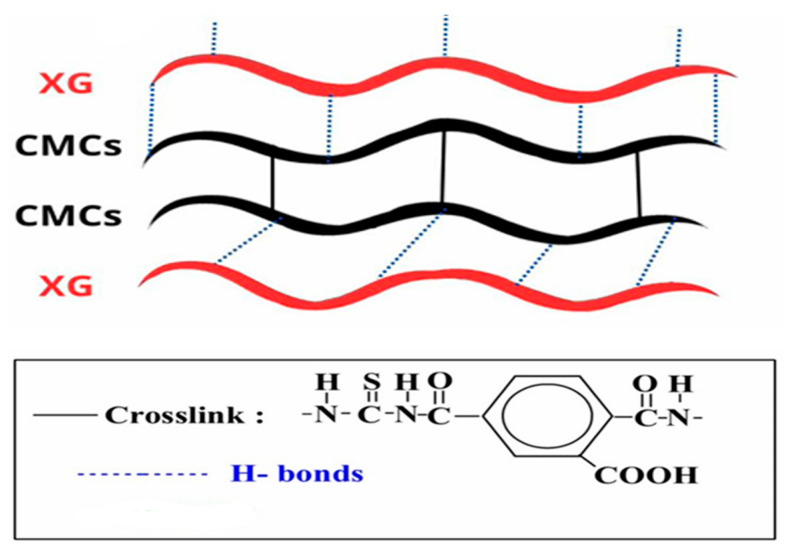
Illustrative depiction of the prepared hydrogel matrix.

CMCs/XG hydrogel was synthesized with a two-step process. The first step included the synthesis of the crosslinker, trimellitic anhydride isothiocyanate (TAI), in accordance with a previously described procedure [38]. In a brief, solid trimellitic anhydride chloride (1.012 g, 4.8 mmol) was gradually added to an equivalent molar ratio of ammonium thiocyanate (0.366 g, 4.8 mmol) suspended in 30 mL of dichloromethane. PEG-400 (1 mL; used as an agent for phase transfer) was added for catalysis, and the reaction mixture was gently stirred at room temperature for 2 h. Then, the by-product, ammonium chloride (formed as a white precipitate), was isolated by filtration to obtain TAI (Figure 2c) as a yellow filtrate. The second step entailed the preparation of CMCs/XG hydrogel as previously described [35]: The aqueous solutions of CMCs (2 g (9.6 mmol), in 100 mL of deionized water) and XG (2 g in 100 mL of deionized water) were thoroughly blended using a mechanical stirrer at 333 ± 5 K for 45 min. Afterwards, the crosslinker (TAI) was gradually added to the produced blend solution and thoroughly stirred at 333 ± 5 K for 4 h, then at room temperature overnight. The TAI-to-CMCs molar ratio was 1:2, respectively. The formed hydrogel was flooded in methyl alcohol for 24 h for dewatering and filtering and dried at 333 ± 5 K to fixed weight. It was yellow in color and was denoted CMCs/XG hydrogel (Figure 3).

### 2.3. Measurements

#### 2.3.1. FTIR Spectrometry

The chemical structural differences between the prepared hydrogel and its pristine components were inspected utilizing an FTIR spectrometer (Model Cary 600 Series, Agilent Technologies, Santa Clara, CA, USA) within the range of wavenumbers from 4000 to 400 cm^−1^.

#### 2.3.2. X-Ray Diffractometry (XRD)

The internal structural variations between the prepared hydrogel and its pristine components were checked by employing a Rigaku Ultima IV-X-ray diffractometer (Tokyo, Japan). The samples in powder form were scanned at diffraction angles from 3° to 90° at 8°/min velocity and room temperature.

#### 2.3.3. Scanning Electron Microscopy (SEM)

The alterations in surface morphology between the prepared hydrogel and its pristine components were observed using an ultrahigh-resolution scanning electron microscope (Schottky Field Emission JEOL 7610F, Tokyo, Japan). The hydrogel and its pristine components were plated with a thin layer of gold, and photographs were taken at 2500× magnification.

#### 2.3.4. Swelling Behavior

The hydrogel (20 mg) was introduced into assorted buffer media (20 mL, pH = 4, 7, and 9) at 293 K and placed in a shaker for various time durations. Then, the swollen hydrogel was extracted and weighed. The swelling capacity was calculated utilizing Equation (1).

% Swelling capacity = [(W_1_ − W_0_)/W_0_] × 100 (1)

where W_1_ and W_0_ are the masses of the swollen and dry hydrogel samples, respectively [39]. The results are given in the form of means ± SDs of three comparable separate tests.

#### 2.3.5. Adsorption Studies

A set of tests was performed to study the adsorption of CIP onto the prepared hydrogel. Hydrogel samples of known weight (10 mg) were placed inside conical flasks holding an aqueous solution of CIP at different concentrations (50–300 mg L^−1^) and were vibrated in a shaker/water bath (80 rpm) at a specific temperature until equilibrium was obtained. Then, the solutions were passed through filter paper (Whatman; 0.45 μm), and the concentrations of CIP were measured by employing a Shimadzu ultraviolet/visible 1601 spectrophotometer (Tokyo, Japan) at 280 nm and quantitively determined with the calibration curves of standard CIP solutions. Adsorption capacity was estimated multiple times, and the average of three comparable results was taken. It is worth mentioning that the same adsorbance of the CIP solution was obtained before and after filtering it, indicating that no CIP adsorption occurred onto the filter paper during filtration.

Adsorption experiments were performed using pH ranging from 2 to 12, adjusted by adding HCl (0.1 N) or NaOH (0.1 N), to study the impact of pH, with the other parameters being kept constant: CIP solution, 10 mL at 50 mg L^−1^ concentration; hydrogel dose, 10 mg; and temperature, 298 K.

Furthermore, various temperatures (293, 303, 313, and 323 K) were set in another batch of experiments to investigate the effect of this parameter, with the other parameters being kept constant: CIP solution, 10 mL at 50 mg L^−1^ concentration; hydrogel dose, 10 mg; and solution pH = 4.

Adsorption tests were also performed by employing various hydrogel doses ranging from 5 to 20 mg to evaluate the influence of this parameter, with the other parameters being kept constant: CIP solution, 10 mL at 50 mg L^−1^ concentration; temperature = 298 K; and solution pH = 4.

Equations (2)–(4) can be utilized for calculating the amount of CIP adsorbed onto the hydrogel.

(2) $$q_{e}=\frac{\left(C_{o}-C_{e}\right)V}{m}$$

(3) $$q_{t}=\frac{\left(C_{o}-C_{t}\right)V}{m}$$

(4) $$\%\;Removal\;efficiency\left(\%\;RE\right)=\frac{C_{o}-C_{e}}{C_{o}}\times 100$$

where the potencies for adsorption (mg g^−1^) are denoted by $q_{e}$ (at equilibrium) and $q_{t}$ (at time *t*). The CIP concentrations (mg L^−1^) are denoted by $C_{o}$ (prior to hydrogel immersion), $C_{e}$ (at equilibrium), and $C_{t}$ (at time *t*). The CIP solution volume (L) and the hydrogel mass (g) are denoted by *V* and *m*, respectively. The results are given in the form of means ± SDs of three comparable separate tests.

#### 2.3.6. Regeneration of Hydrogel for Reuse

CIP was removed from the hydrogel by washing the latter with distilled H_2_O to eliminate non-adsorbed CIP; then, 10 mg of the resulting hydrogel was soaked in HCl (10 mL, 0.1 M) at room temperature overnight. The amount of CIP desorbed from the hydrogel was determined utilizing Equation (5).

% CIP desorption = *q_d_*/*q_a_* × 100 (5)

where the quantities of CIP desorbed from and adsorbed onto the hydrogel are denoted by *q_d_* and *q_a_* (mg g^−1^), respectively [40].

## 3. Results and Discussion

### 3.1. Hydrogel Synthesis

A hydrogel based on a blend of CMCs and XG in an equivalent-weight ratio was synthesized with a chemical crosslinker, TAI (Figure 2c). In addition, the formation of H-bonds between XG and CMCs indicated that a physical crosslinking process also occurred (Figure 3). The chemical crosslinking procedure was obtained by allowing the 1^ry^ amino groups of CMCs to interact with both groups of the crosslinker (TAI) (anhydride and isothiocyanate), as confirmed by the insolubility of the produced crosslinked CMCs at all pH values. On the other hand, XG did not show any ability to crosslink with TAI due to weak OH group activity in XG compared with the NH_2_ groups in CMCs, as evidenced by identical FTIR spectra of XG before and after its treatment with TAI.

### 3.2. Characterization of Hydrogel

#### 3.2.1. FTIR Spectrometry

**Figure 4:**
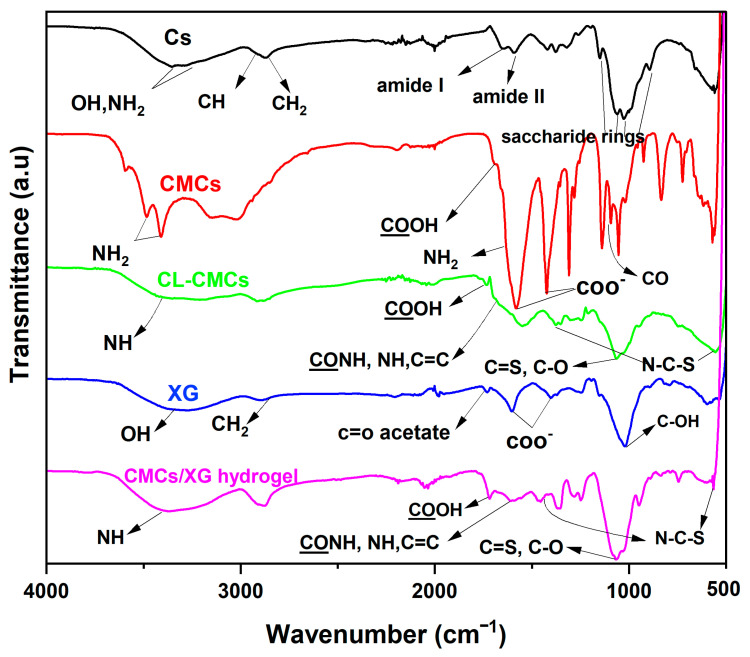
FTIR spectra of the prepared hydrogel as well as its pristine components.

As shown in Figure 4, Cs shows distinct stretching vibration bands at (1) 3600–3000 cm^−1^ (wide and intense), due to H-bonded −OH and −NH_2_ groups, with the doublet band of the latter appearing at 3354 and 3290 cm^−1^; (2) 2916 and 2872 cm^−1^, attributed to C−H (asymmetric and symmetric); (3) 1654 (amide I) and 1591 cm^−1^ (amide II) (weak), confirming the highly deacetylated grade of chitosan; and (4) 1150, 1060, 1026, and 894 cm^−1^, assigned to the nuclei of saccharide [41].

In comparison with Cs, CMCs (Figure 4) possesses similar bands, particularly the double one at 3479 and 3409 cm^−1^, indicating that the −NH_2_ groups were not affected by Cs treatment and confirming the occurrence of the reaction of carboxymethylation selectively at −OH at C6. Moreover, the CMCs spectrum shows new stretching vibration bands at (1) 1700 cm^−1^, attributed to **CO**OH; (2) 1419 cm^−1^ (strong), assigned to carboxylic acid groups (symmetrical); and (3) 1583 cm^−1^, ascribed to carboxylate groups (asymmetric). The latter and the amino deforming vibration at 1598 cm^−1^ overlap, forming an intense band. In addition, a band corresponding to C−O of sec −OH appears stronger and shifted to a longer wavenumber of 1100 cm^−1^ [42].

Figure 4 also shows the spectrum of crosslinked CMCs (CL-CMCs), in which all the bands of CMCs are present, except for the doublet band of −NH_2_ groups, which is replaced by a single band at 3434 cm^−1^. The spectrum also displays a new band at 1729 cm^−1^, corresponding to the crosslinkages of −**CO**OH. The band attributed to CO**OH** overlaps with the N−H band at 3434 cm^−1^. The bands of CONH, sec-amide, and aromatic C=C overlap, resulting in a band at 1650 cm^−1^. In addition, the bands of C=S groups and C−O overlap, forming a strong, wide band at 1063 cm^−1^. Two new bands ascribed to N−C−S groups appear at 422 and 592 cm^−1^ [31,32]. Taken together, these results indicate the successful crosslinking of CMCs.

The spectrum of XG (Figure 4) presents characteristic stretching vibration bands at (1) 3430 cm^−1^ (wide), corresponding to H−bonded O−H groups; (2) 2896 cm^−1^, attributed to C−H groups; (3) 1730 cm^−1^ (weak), assigned to the C=O of acetate groups; (4) 1625 and 1426 cm^−1^ (asymmetric and symmetric, respectively), ascribed to carboxylate groups; and (5) 1041 cm^−1^, due to C–OH groups [43].

The spectrum of the CMCs/XG hydrogel (Figure 4) displays the replacement of the 1^ry^ NH_2_ doublet band of CMCs with a single band at 3368 cm^−1^ ascribed to N–H groups, indicating the completion of the reaction between the –NH_2_ of CMCs and TAI. The band of **CO**OH groups appears at 1718 cm^−1^ and overlaps with that of the N–H groups. In addition, the bands of aromatic C=C, N–H, and CONH groups overlap, creating a wide band at 1650 cm^−1^. The overlapping of C=S and C–O bands produced a strong, wide band at 1058 cm^−1^, and the bands at 1426 and 567 cm^−1^ are ascribed to the bending vibration of N–C–S groups [44].

#### 3.2.2. X-Ray Diffractometry (XRD)

**Figure 5:**
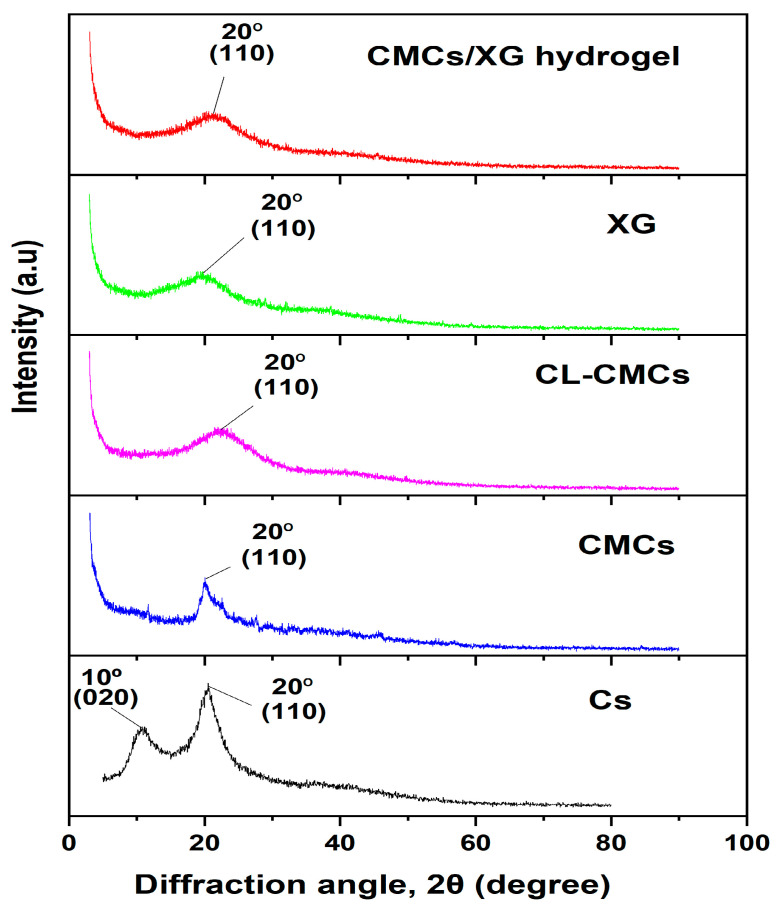
XRD patterns of the prepared hydrogel and its pristine components.

The differences between the interior structure of the hydrogel and its base ingredients were inspected using XRD, as shown in Figure 5. Pristine Cs possesses two characteristic peaks: the first peak appears near the diffraction angle (2θ) = 10° and corresponds to its amorphous fraction, and the second one manifests near 2θ = 20° and is attributed to its crystalline fraction [45]. The abundance of polar groups of Cs (–OH and –NH_2_) is considered responsible for the formation of numerous intra- and interchain H-bonds, which in turn are accountable for the XRD pattern of Cs. CMCs shows less crystallinity, as evidenced by its XRD pattern, which lost the first peak and retained the second, but with lower intensity relative to the corresponding peak in the parent Cs (Figure 5). The crosslinking of CMCs (CL-CMCs) led to a remarkable reduction in its H-bonds due to the modification of its amino groups. Further, the addition of TAI resulted in the separation of its chains, which increased the amorphous portion while decreasing the crystalline region. These conclusions are evidenced by an appreciable widening accompanied by decreased intensity of the second peak (Figure 5). The parent XG displayed a weakly wide peak at 2θ near 20° (Figure 5), indicative of its amorphous characteristics [46]. Thus, the hydrogel is expected to exhibit a single peak at 2θ near 20° (Figure 5), confirming its amorphous nature.

#### 3.2.3. Scanning Electron Microscopy (SEM)

**Figure 6:**
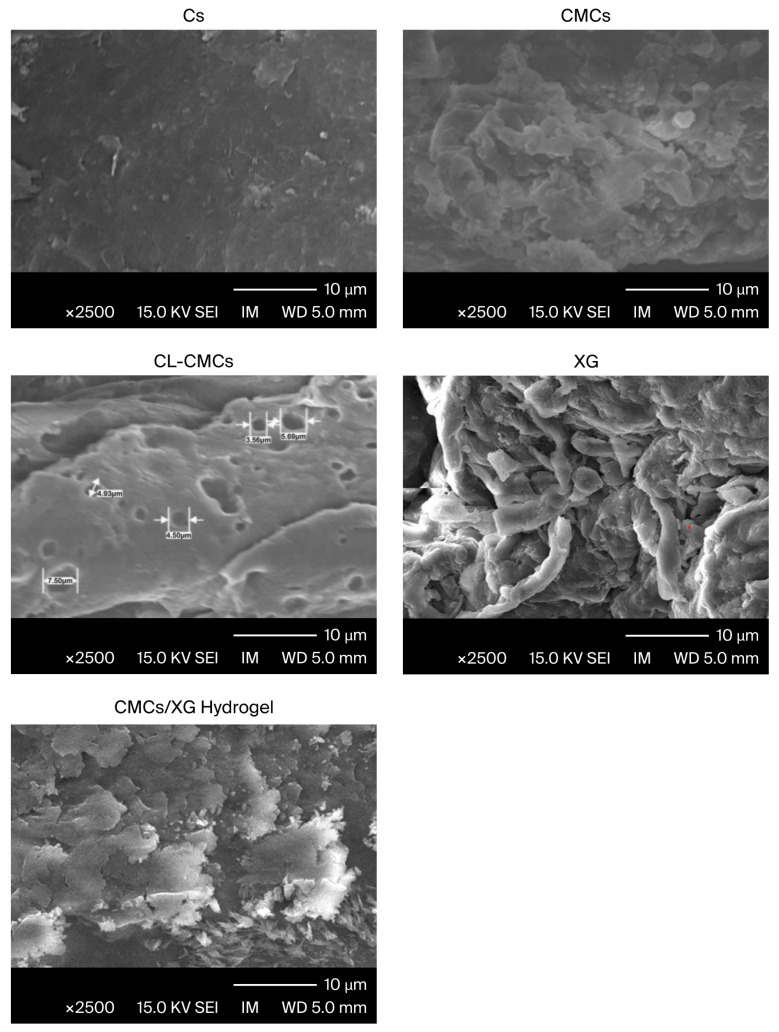
SEM micrographs of the prepared hydrogel and its pristine components.

The topography of the surfaces of the prepared hydrogel and its pristine components was investigated using SEM, and the micrographs are displayed in Figure 6. The modification of Cs altered its smooth outside surface, as indicated by the coarse CMCs topography with numerous lumps. This was due to the incorporation of carboxymethyl groups into Cs. The CL−CMCs topography showed a considerably pored surface, where pores act as regions for the entry of water and as sites of interaction between both functional polar groups of CL−CMCs with exterior stimuli. Long TAI linkages resulted in the formation of big pores, permitting the imbibition of a big quantity of fluids and enhancing the interactions between the added compounds and CL−CMCs. XG showed a fibrous surface. The CMCs/XG hydrogel had a coarse surface with many flutings and contained numerous pores resulting from crosslinking networks. The uniformly distributed flutings observed indicate the effective completion of the crosslinking process. Upon the addition of the crosslinker, the number of H-bonds between the polymeric chains is considerably diminished, leading to chain separation and the formation of a significantly open matrix and a larger surface area. This facilitates the permeation of fluids inside the hydrogel and the formation of sites for interaction between the hydrogel’s polar groups and external stimuli.

#### 3.2.4. Swelling Behavior

**Figure 7:**
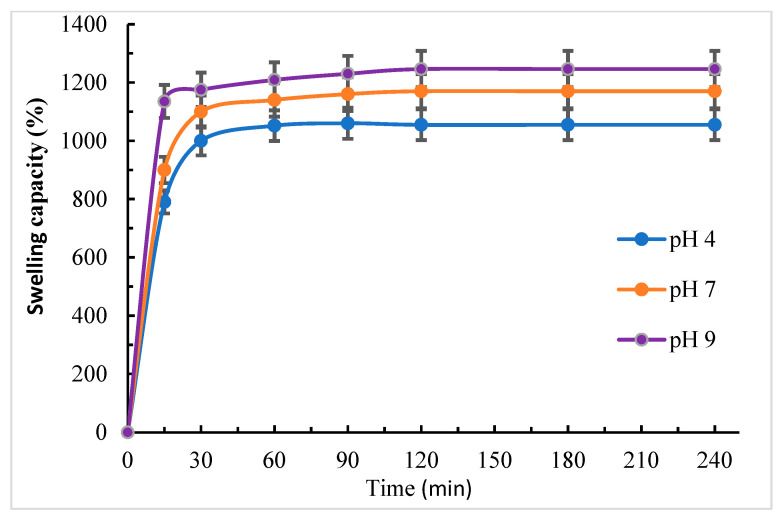
Swelling performance of the prepared hydrogel under various conditions (hydrogel mass = 20 mg; buffer solutions (20 mL) at pH = 4, 7, and 9; temperature = 298 K; and various time durations).

Figure 7 presents data on the swelling performance of the prepared hydrogel at 298 K in solutions having different pH values (4, 7, and 9), for various time durations required to reach equilibrium. It can be noted that under all conditions, the swelling of the hydrogel sharply increased until 120 min and then settled.

The swelling performance of the hydrogel is influenced by the medium pH due to the former’s numerous polar groups, including hydroxyl, carboxylic, thiourea, and amide groups, in addition to the spaces resulting from the crosslinking reaction. Therefore, it serves as a polycationic or polyanionic hydrogel depending on the medium’s pH.

In an alkaline medium (pH 9), the protons of the carboxylic groups are removed to yield the corresponding carboxylate anion-bearing negative charges, while the other functional groups (amino, carbonyl, and thiocarbonyl) are unaltered, generating an anionic-charged hydrogel.

Swelling efficiency depends on variations in osmosis and repulsion interaction between carboxylate ions. Thus, the water that enters the hydrogel matrix bonds with the carboxylate ions. The increase in hydrogel swelling capacity in an alkaline medium in comparison with a neutral one is due to the conversion of carboxylic groups into carboxylate ions.

In an acidic medium (pH = 4), the polar groups (amino, carbonyl, and thiocarbonyl) are protonated to NH_2_^+^, C=SH^+^, and C=OH^+^ cations, while the −COOH groups are unaltered, forming a cationic-charged hydrogel. The generated cations and consequently hydrogel chains repel each other. Further, inside the hydrogel matrix, an increment in mobile ions boosts the ionic difference between inside and outside the matrix: this is the driving force of its swelling capacity.

The swelling capacity of the hydrogel in basic media is higher than that in neutral or acidic media. This can be attributed to the existence of acidic groups (−COOH) in both CMCs moieties and TAI linkages, which easily transform into the corresponding -COO^−^ ions. The latter repel each other inside the hydrogel matrix, promoting osmosis and facilitating the entry of water into the hydrogel (Figure 7).

### 3.3. Adsorption Optimization

#### 3.3.1. Impact of Medium pH

The sorption of CIP onto the hydrogel is markedly influenced by the medium pH owing to various CIP species, in addition to different charges on the hydrogel surface.

**Figure 8:**
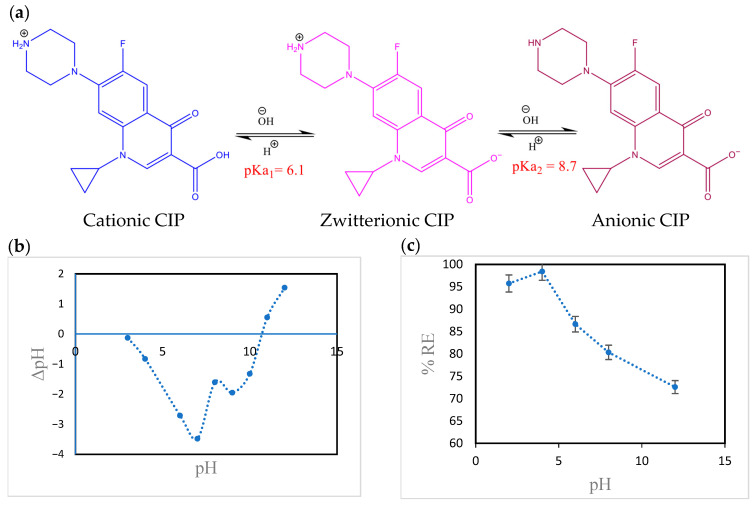
pH-based CIP sorption mechanism: (**a**) CIP ionization, (**b**) pHzpc measurements of the hydrogel, and (**c**) pH-based CIP sorption.

CIP molecules are found in three distinguished states according to medium pH: The cationic state (CIP^+^) occurs when the medium pH is lower than pK_a1_ (6.1) due to the protonation of the -NH group of the piperazine ring, as shown in Figure 8a. The anionic state (CIP^−^) occurs when the medium pH is higher than pK_a2_ (8.7), owing to the deprotonation of the -COOH group. When the medium pH falls between pK_a1_ and pK_a2_, the molecules of CIP turn into zwitterions (CIP^±^). This behavior is ascribed to the protonation of the -NH group at the piperazine nucleus and the deprotonation of the -COOH group in CIP [47].

Measurements of pH_zpc_ elucidate the influence of the medium’s pH on the protonation and deprotonation of the hydrogel surface. The pH_zpc_ value of the hydrogel was found to be 10.5 (Figure 8b). The charges of the hydrogel surface were positive at pH < pH_zpc_ and negative at pH > pH_zpc_.

Figure 8c illustrates the impact of medium pH on the sorption of CIP onto the hydrogel. It is noticeable that CIP removal slightly improved when the solution pH (ranging from 2 to 4) was less than pK_a1_, indicating that CIP existed in its cationic state. Further, the pH_zpc_ results indicate the development of positive charges on the hydrogel matrix. Thus, the mechanism of CIP sorption mostly comprises cation exchange, in addition to CIP molecules/hydrogel interlayer complexation. A decrease in CIP sorption was observed when pK_a1_ < pH < pK_a2_. In this pH range, the surface of the hydrogel carries positive charges, and the CIP molecules are zwitterions. Consequently, high CIP sorption is achieved via a cation exchange mechanism between the positively charged groups on both the hydrogel and the CIP zwitterions [48], together with the mechanism of electrostatic attraction between the negatively charged groups on the CIP zwitterions and the positively charged groups on the hydrogel. A further increase in solution pH to values higher than pK_a2_ led to a decrease in CIP sorption. This may be ascribed to the repulsion forces between anionic CIP and the negative charges on the hydrogel surface. This is consistent with research results on CIP removal with schorl [20] and ciprofloxacin adsorption on kaolinite [18].

#### 3.3.2. Impact of Hydrogel Dose

**Figure 9:**
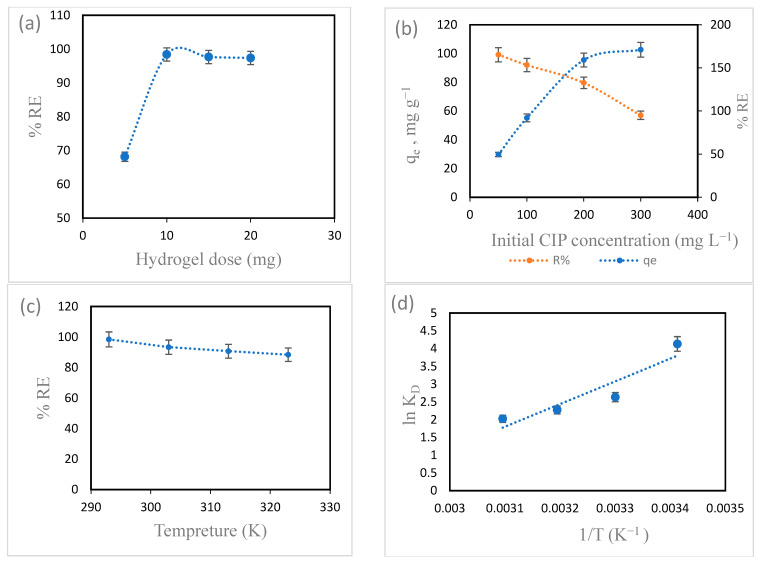
Effects of (**a**) hydrogel dosage, (**b**) initial CIP concentration, (**c**) temperature, and (**d**) lnK_D_ versus 1/T on CIP sorption (volume of CIP solution = 10 mL; hydrogel dose = 10 mg).

To optimize the quantity of hydrogel required for achieving the greatest CIP sorption, it is necessary to evaluate the impact of hydrogel dose on CIP removal performance and CIP sorption capability. The plots of the RE (%) of CIP versus hydrogel dose are shown in Figure 9a. RE (%) improved with an increment in the weight of the hydrogel from 5 mg (% RE = 68.14%) to 10 mg (% RE = 98.41%). This is due to the increase in the sites available for binding. Further increase in hydrogel dose did not show appreciable increases in RE (%). Thus, the optimum % RE was obtained at the hydrogel dose of 10 mg, which was selected for further investigations. This is in agreement with a study on the removal of CIP using CIP CeO_2_-NPs [24].

#### 3.3.3. Impact of CIP Concentration 

Initial pollutant concentration is considered to be a prime factor influencing the sorption process. We explored the effect of initial CIP concentrations ranging between 50 and 300 mg L^−1^ on the adsorption process. As shown in Figure 9b, RE (%) is inversely proportional to initial CIP concentration: it decreased from 99.17 to 57.03% with the increase in initial CIP concentration from 50 to 300 mg L^−1^. This is because the number of active sites available for adsorption remains constant since the quantity of hydrogel is constant. Thus, with the increase in pollutant concentration, the number of pollutant molecules increased, saturating the available adsorption sites, which resulted in a reduction in RE (%) [49]. This is supported by previous studies where chitosan-based zeolite composites and chitosan-based magnetic nanocomposites were used to remove CIP and tetracycline, respectively; the authors reported that the removal efficiency was reduced with the increase in the concentrations of these antibiotics [50,51].

#### 3.3.4. Impact of Temperature

The impact of different temperatures, ranging between 293 and 323 K, on CIP adsorption onto the prepared hydrogel was also studied. As illustrated in Figure 9c, with the increase in the temperature of the adsorption process from 293 to 323 K, RE (%) decreased from 98.41 to 88.34%. This indicates that the adsorption process is exothermic.

At high temperatures, the electrostatic interaction of CIP with the hydrogel decreases, which consequently decreases the CIP elimination rate. Moreover, CIP solubility increases with temperature; thus, the interaction forces between CIP and water become more robust than those between CIP and the hydrogel surface. These findings are in good agreement with previously reported results on the absorption efficiency of magnetic-chitosan nanocomposites in the removal of the antibiotic tetracycline from aqueous solutions [51].

#### 3.3.5. Thermodynamic Study

The thermodynamic parameters of CIP removal, i.e., changes in Gibbs free energy (∆G°, KJ mol^−1^), enthalpy (∆H°, KJ mol^−1^), and entropy (∆S°, J K^−1^ mol), can be calculated utilizing Equations (6)–(8).

(6) $$∆G{}^{\circ}=-RT\ln K_{D}$$

(7) $${lnK}_{D}=-\frac{∆H{}^{\circ}}{RT}-\frac{∆S{}^{\circ}}{R}$$

(8) $$K_{D}=\frac{q_{e}}{C_{e}}$$

where *K_D_* and *R* (8.314, J/mol K) are the equilibrium constant and the global gas constant, respectively.

The data in Table 1 show that CIP adsorption onto the prepared hydrogel occurred spontaneously and favorably because of the negative values of ∆G°. It can be noted that the entropy decreased as the temperature increased. Finally, the negative value of ∆H° indicates that the CIP adsorption process is exothermic.

Furthermore, the exothermic nature of this process is more desirable at lower temperatures owing to the decreased values of ∆G°. For the latter, we recorded values between 5.439 and 10.055 KJ mol^−1^, which are indicative of the physical nature of CIP adsorption onto the prepared hydrogel. Furthermore, the negative ∆S° value indicates a decrease in the random interaction between the hydrogel surface and CIP as the temperature increased [52].

### 3.4. Adsorption Kinetics

Contact time is considered to be one of the most significant parameters in determining the time of equilibrium; its study also provides insights into the rate and mechanism of the sorption process. Experiments were designed with the following settings: various contact times, 1–180 min; CIP, 50 mL at 50 mg L^−1^; hydrogel dose, 0.01 mg L^−1^; and temperature, 298 K.

**Figure 10:**
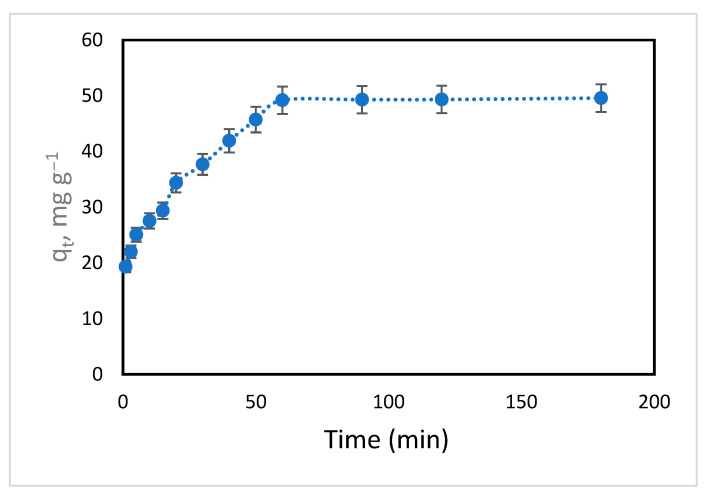
Impact of contact time on CIP adsorption onto hydrogel (initial CIP concentration = 50 mg L^−1^; temperature = 293 K; volume of CIP solution = 50 mL; hydrogel dose = 10 mg).

Figure 10 shows the plot of contact time against adsorption capacity. The latter was significantly boosted during the initial 60 min (rapid adsorption), achieving 99.17% RE and a *q_t_* of 49.21 mg g^−1^ from the CIP solution; then, it reached equilibrium. Thus, the 180 min contact time was chosen for further testing.

**Figure 11:**
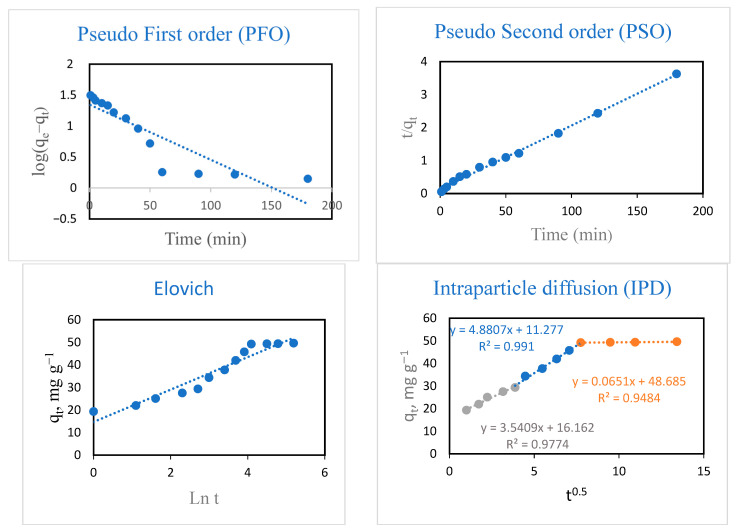
Kinetic modeling of CIP sorption onto the hydrogel (initial CIP concentration = 50 mg L^−1^; temperature = 293 K; volume of CIP solution = 50 mL; and hydrogel dose = 10 mg).

The experimental data of CIP adsorption onto the hydrogel were fitted with pseudo-first-order (PFO), pseudo-second-order (PSO), Elovich, and intraparticle diffusion (IPD) kinetic models (Figure 11). The equations and the calculated parameters of the kinetic models are shown in Table 2. It was found that the PSO model effectively describes the CIP adsorption process. This can be ascribed to the highest value of *R*^2^ (0.997), as well as the close match of the calculated (*q_e,cal._*, 51.81 mg g^−1^) and experimental (*q_e,exp._*, 49.59 mg g^−1^) adsorption capacity (Table 2). This suggests that the adsorption process is chemical in nature, with adsorption capability depending on the number of reactive binding centers on the hydrogel [21,23].

In accordance with the Weber–Morris theory, the mechanism of adsorption can be fitted with an IPD model if the linear plotting curve passes through the origin; in this case, the prime rate-dominant step is IPD. Otherwise, in addition to IPD, other mechanisms could also be involved [53,54]. There are three linear steps in the plot of the IPD of the adsorption process. The transfer of CIP from the feed medium to the surface of montmorillonite was interpreted as consisting of three consecutive steps: (1) external (surface) prevalence, where CIP molecules transfer from the medium to the exterior surface of montmorillonite; (2) internal (intraparticle) prevalence, where CIP molecules transfer from the outer surface of montmorillonite to its inner portion; and (3) the adsorption of diffused CIP onto montmorillonite active binding sites [23]. In this study, the IPD curve of the hydrogel shows a negative pass via the origin in the experimental results, indicating that IPD is not the only rate-dominant step, with other mechanisms contributing to the adsorption process.

### 3.5. Adsorption Isotherm

**Figure 12:**
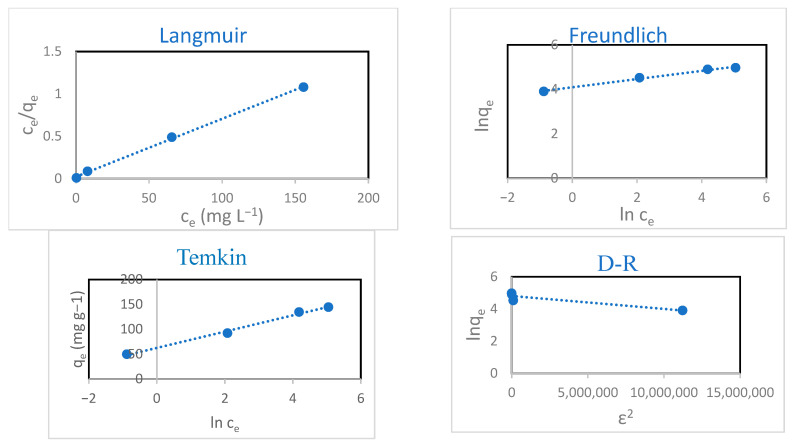
Isotherm modeling of CIP adsorption onto hydrogel (time = 48 h, temperature = 293 K, volume of CIP solution = 10 mL, and hydrogel dose = 10 mg).

The plots of the results of the hydrogel adsorption isotherms obtained using various concentrations of CIP (50–300 mg L^−1^) are illustrated in Figure 12. It can be seen that as the CIP concentration increased, so did the hydrogel adsorption capacity, until saturation was progressively reached. The experimental outcomes are presented utilizing the nonlinear forms of the isotherm models Langmuir, Freundlich, Temkin, and Dubinin–Radushkevich (D-R) in an effort to understand the underlying adsorption mechanism. Table 3 summarizes the equations, constants, calculated values of the parameters, and regression coefficients of all the applied models. The model parameters in Table 3 confirm that the Langmuir model was the most consistent with hydrogel adsorption (*R*^2^ = 0.999). Thus, adsorption occurs homogeneously on the hydrogel to form a CIP monolayer. Moreover, based on the Langmuir model, adsorption favorability was assessed with the dimensionless equilibrium parameter (*R_L_*). The hydrogel showed *R_L_* values between 0 and 1: the adsorption process was favorable, indicating a strong interaction between the hydrogel and CIP. The maximum monolayer adsorption capacity (*q_max_*) was found to be 147.059 mg g^−1^.

### 3.6. Analysis of Sorption Capacity of CIP onto Different Adsorbents

In Table 4, we compare the maximum CIP monolayer sorption capacity (*q_max_*) of the hydrogel with those of other previously reported adsorbents based on Cs and XG to determine whether the investigated hydrogel has higher or lower capacity than the other reported options. The studied hydrogel showed good adsorption capacity owing to its high functionality, porosity, and swelling capacity.

### 3.7. Cycling Test

**Figure 13:**
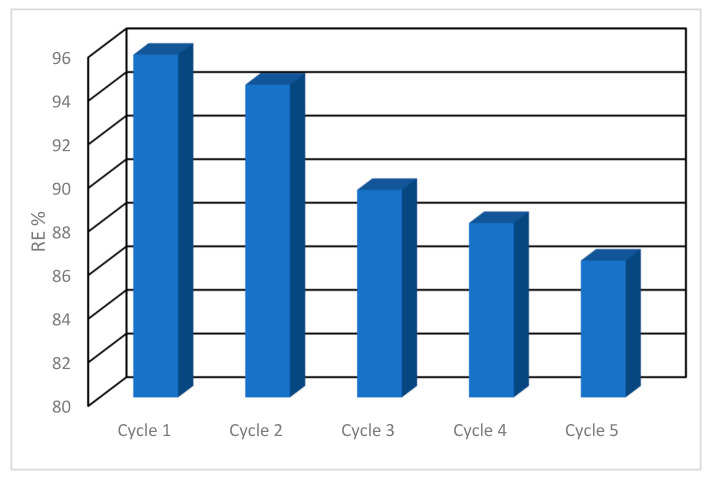
CIP removal efficiency over five consecutive cycles using hydrogel.

The recycling capability of adsorbents has considerable significance in achieving cost-effective water remediation. The restored hydrogel was restored for five consecutive cycles via adsorption/desorption processes using 0.1 M HCl for the desorption step. The results show that CIP adsorption slightly decreased from 95.72 to 94.34, 89.52, 88, and 86.28% during these five cycles (Figure 13). This could be attributed to failure to release all active adsorption sites during the desorption process. The experimental outcomes confirm that the investigated hydrogel can be efficiently restored for reuse via adsorption/desorption processes.

## 4. Conclusions

XG was blended with CMCs in an equivalent-weight ratio, followed by crosslinking with TAI at a concentration depending on the CMCs concentration in the blend, to produce CMCs/XG hydrogel. The parent CMCs was also crosslinked alone with TAI to produce CL-CMCs. The chemical, inner, and morphological structures of the CMCs/XG hydrogel were compared to those of its pure components using FTIR spectroscopy, XRD diffractometry, and SEM observation. The sorption of CIP onto the hydrogel was markedly influenced by the pH of the medium, dose of the hydrogel, initial CIP concentration, and temperature. CIP adsorption onto the hydrogel proceeded spontaneously and favorably and was exothermic in nature. The PSO model was found to effectively fit the CIP adsorption process. Thus, it is concluded that CIP adsorption includes physical and chemical adsorption processes. The Langmuir model was the most consistent with the investigated process, indicating that the hydrogel absorbs homogeneously to form a CIP monolayer. The maximum monolayer adsorption capacity (*q_max_*) was found to be 147.059 mg g^−1^. The investigated hydrogel can be efficiently restored for reuse via adsorption/desorption processes.

## Figures and Tables

**Table 1:** Thermodynamic parameters of CIP sorption onto the hydrogel.

Temperature, K	∆G°, KJ mol^−1^	∆H°, KJ mol^−1^	∆S°, J K^−1^ mol
293	−10.055	−53.057	−149.494
303	−6.639		
313	−5.924		
323	−5.439		

**Table 2:** Parameters of PFO, PSO, Elovich, and IPD models for CIP adsorption process.

Linear Kinetic Model	Equation	Parameter	Value
PFO	$\log\left(q_{e}-q_{t}\right)=\log q_{e}-\frac{k_{1}}{2.303}t$	*k*_1_ (min^−1^)	0.049515
		*q_e,cal._* (mg g^−1^)	4.700176
		*q_e,exp._* (mg g^−1^)	49.59
		*R* ^2^	0.954
PSO	$\frac{t}{q_{t}}=\frac{1}{k_{2}q_{e}^{2}}+\frac{t}{q_{e}}$	*K*_2_ (g mg^−1^ min^−1^)	0.002755
		*q_e,cal._* (mg g^−1^)	51.81347
		*R* ^2^	0.997
Elovich	*q_t_ =*$\frac{1}{\beta }$ ln(*αβ*) + $\frac{1}{\beta }$ ln *t*	*α* (mg g^−1^ min^−1^)	55.94023
		*β* (g mg^−1^)	0.139431
		*R* ^2^	0.928
IPD	*q_t_* = (*k_int_ t* ^1/2^) + *C*	*K*_1_ (mg g^−1^ h^−0.5^)	3.541
		*C*_1_	16.16
		*R* ^2^	0.977
		*K*_2_	4.881
		*C*_2_	11.28
		*R* ^2^	0.991
		*K*_3_	48.685
		*C*_3_	0.993
		*R* ^2^	0.948

**Table 3:** Equations and parameters of isotherm models of CIP adsorption onto the hydrogel.

Isotherm Model	Equation	Parameter	Value
Langmuir isotherm	$\frac{C_{e}}{q_{e}}=\frac{1}{q_{max}K_{L}}+\frac{C_{e}}{q_{max}}$ $R_{L}=\frac{1}{\left(1+K_{L}C_{o}\right)}$	*K_L_* (mg g^−1^)	0.289
		*q_max_* (mg g^−1^)	147.059
		*R_L_*	0.065 − 0.011
		*R* ^2^	0.999
Freundlich isotherm	$\ln q_{e}=\ln K_{F}+\frac{1}{n}{lnC}_{e}$	*K_F_* (mg g^−1^)	59.979
		1/*n*	0.184
		*R* ^2^	0.991
Temkin isotherm	*q*_*e*_ = *B*_*T*_ln*K_T_* + *B_T_*ln *C*_*e*_	*B*	16.372
		*K_T_* (L g^−1^)	45.059
		*R* ^2^	0.994
Dubinin–Radushkevich (D-R) isotherm	ln *q_e_* = ln (*q_m_*) − *βε*^2^ *ε* = *RT* $\ln(1+\frac{1}{C_{e}})$ *E* = ($\frac{1}{{\left(2\beta \right)}^{0.5}}$)	*β* (mol^2^ KjKJ^−2^)	$8\times 10^{-8}$
		*q_s_* (mg g^−1^)	121.754
		*ε*	0.006
		*R* ^2^	0.843

**Table 4:** CIP adsorption capacity onto different chitosan and xanthan gum-based adsorbing materials.

Adsorbing Material	Adsorption Capacity *q_max_* (mg g^−1^)	pH	Reference
Cs/kaolin/Fe_3_O_4_	47.85	6	[55]
Trifunctional chitosan-EDTA-β-cyclodextrin	49.37	4–6	[56]
Cs-grafted SiO_2_/Fe_3_O_4_	52.14	12	[57]
Chitosan/biochar hydrogel beads	76.00	3	[58]
Magnetic activated carbon/chitosan composite	90.01	-	[59]
Magnetite-imprinted chitosan nanocomposite	142.85	6	[60]
Humic acid-coated biochar and chitosan	154.89	8	[61]
CoFe_2_O_4_/activated carbon@ chitosan	188.00	5	[62]
MIL53-NH_2_/xanthan gum/Fe_3_O_4_	237.56	3–11	[63]
The investigated hydrogel	147.06	0.05	Present study

## Data Availability

The original contributions presented in this study are included in the article. Further inquiries can be directed to the corresponding authors.
